# Comparative Study of Fig Volatile Compounds Using Headspace Solid-Phase Microextraction-Gas Chromatography/Mass Spectrometry: Effects of Cultivars and Ripening Stages

**DOI:** 10.3389/fpls.2021.667809

**Published:** 2021-07-02

**Authors:** Kahina Zidi, Djamel Edine Kati, Mostapha Bachir-bey, Manon Genva, Marie-Laure Fauconnier

**Affiliations:** ^1^Laboratoire de Biochimie Appliquée, Faculté des Sciences de la Nature et de la Vie, Université de Bejaia, Bejaia, Algeria; ^2^Laboratory of Chemistry of Natural Molecules, Gembloux Agro-Bio Tech, University of Liège, Gembloux, Belgium

**Keywords:** *Ficus carica* L., volatile organic compounds, ripening stages, head-space solid-phase microextraction, aroma, agrobiodiversity

## Abstract

Aroma is one of the essential parameters that determine fruit quality. It is also an important feature of varietal characterization and so valuable for agro-biodiversity identification and preservation. In order to characterize changes in the aroma fingerprint through fig development, the main objective of the present research was to study the volatile organic compound (VOC) profiles of figs (*Ficus carica* L.) from three cultivars, Taamriwthe (TH), Azegzaw (AZ), and Averkane (AV), at three ripening stages (unripe, ripe, and fully ripe). Analyses was performed using Headspace Solid-phase Microextraction and gas chromatography coupled with mass spectrometry. Results revealed the presence of 29 compounds that were grouped into different chemical classes. Aldehydes comprised the most abundant VOCs identified in all the studied figs, while alcohols, ketones, and terpenes comprised the minor compounds found in TH, AZ, and AV figs, respectively. Different aroma descriptors were identified throughout the ripening stages of figs; fruity and green aromas were dominant in all cultivars, while a fatty aroma scarcely occurred in figs. A gallery plot representation demonstrated that certain VOCs differentiate the studied cultivars and the different ripening stages of figs. Principal component analysis findings demonstrated characteristic VOCs of distinct ripening stages and cultivars, those VOCs can be used as fingerprints to distinguish different cultivars and/or ripening stages.

## Introduction

Figs (*Ficus carica* L.), which have always been appreciated for their sweet taste and high nutritional value, are an essential constituent of the Mediterranean diet alongside olive oil, cereals, and vegetables. The global fig production is around 1 million tons per year, with Turkey being the largest producer, followed by Egypt, Morocco, and Algeria (FAO, 2018). Figs are characterized by an inflorescence called a syconium and are rich in minerals, vitamins, and fiber. They are also cholesterol and fat-free (Solomon et al., 2006; Palmeira et al., 2019) and have antioxidant, antimicrobial, antispasmodic, and anticancer properties (Chawla et al., 2012; Barolo et al., 2014; Palmeira et al., 2019). Figs are also an important source of multiple bioactive compounds that have several uses in nutrition and therapy (Palmeira et al., 2019). They are consumed fresh, dried, or transformed into numerous other products such as jams, beverages, and confectionery (Zidi et al., 2020).

Several changes occur during fruit ripening, including physiological, biochemical, and organoleptic modifications. As examples, ethylene production increases, chlorophyll is degraded, anthocyanin content changes, and flavor and aroma modifications are observed throughout the ripening process. Also, a degradation of the cell wall structure during fruit ripening leads to the solubilization of polysaccharides such as pectin and cellulose and induces an increase in fruit sweetness (Prasanna et al., 2007).

Volatile organic compounds (VOCs) are involved in a wide range of functions. For example, VOCs contribute to fruit aroma and to protection against herbivores, microbial growth, and abiotic stress (Schwab et al., 2008). In addition, these compounds attract specific fig pollinators, such as *Blastophaga psenes*, to ensure flower pollination, enabling the development and growth of the fruit (Ware et al., 1993). Fruit VOCs also attract animals that contribute to seed dissemination. Volatile organic compounds that contribute to fruit aroma and flavor are generally present in both free and bound forms. The free VOCs directly contribute to fruit aroma while the bound form is considered to be a non-volatile precursor linked by a β-glycosidic linkage to mono or disaccharides (Hjelmeland and Ebeler, 2015). VOCs are influenced by fruit growth and ripening stages, leading to qualitative and quantitative changes (Iban et al., 2000), which are closely related to the release of VOCs that are often sequestered in glycosylated form (Chen et al., 2021). Thus, an aroma pattern might be used as a marker to discriminate between fruit maturity stages, but it can also be used to identify different fruit origins (genetic or geographic) (Khalil et al., 2017).

Even though figs are one of the oldest cultivated fruits, the changes that occur during the ripening process remain poorly studied. The most widespread methods of fig characterization are phenotypic and genotypic analyses. However, metabolomics, which considers secondary metabolites as the final stage of plant physiological response in a given environment, may be a more appropriate approach. In this context, this study aims to investigate the natural dynamics of the VOC profile throughout the figs' maturation and to demonstrate changes in the key aroma compounds between cultivars during the ripening stages. We thus developed a performant method to analyze fig VOCs, as this fruit emits low quantities of VOCs. Among the different methods tested, Headspace Solid-phase Microextraction (HS-SPME) was chosen for its high sensitivity and extraction efficiency (Elmore et al., 2000; Merkle et al., 2015). In the present study, Principal Component Analysis (PCA) was also conducted in order to determine the VOCs that characterize each cultivar, as well as those determining each ripening stage (unripe, ripe, and fully ripe).

## Materials and Methods

### Plant Material

**Figure 1:**
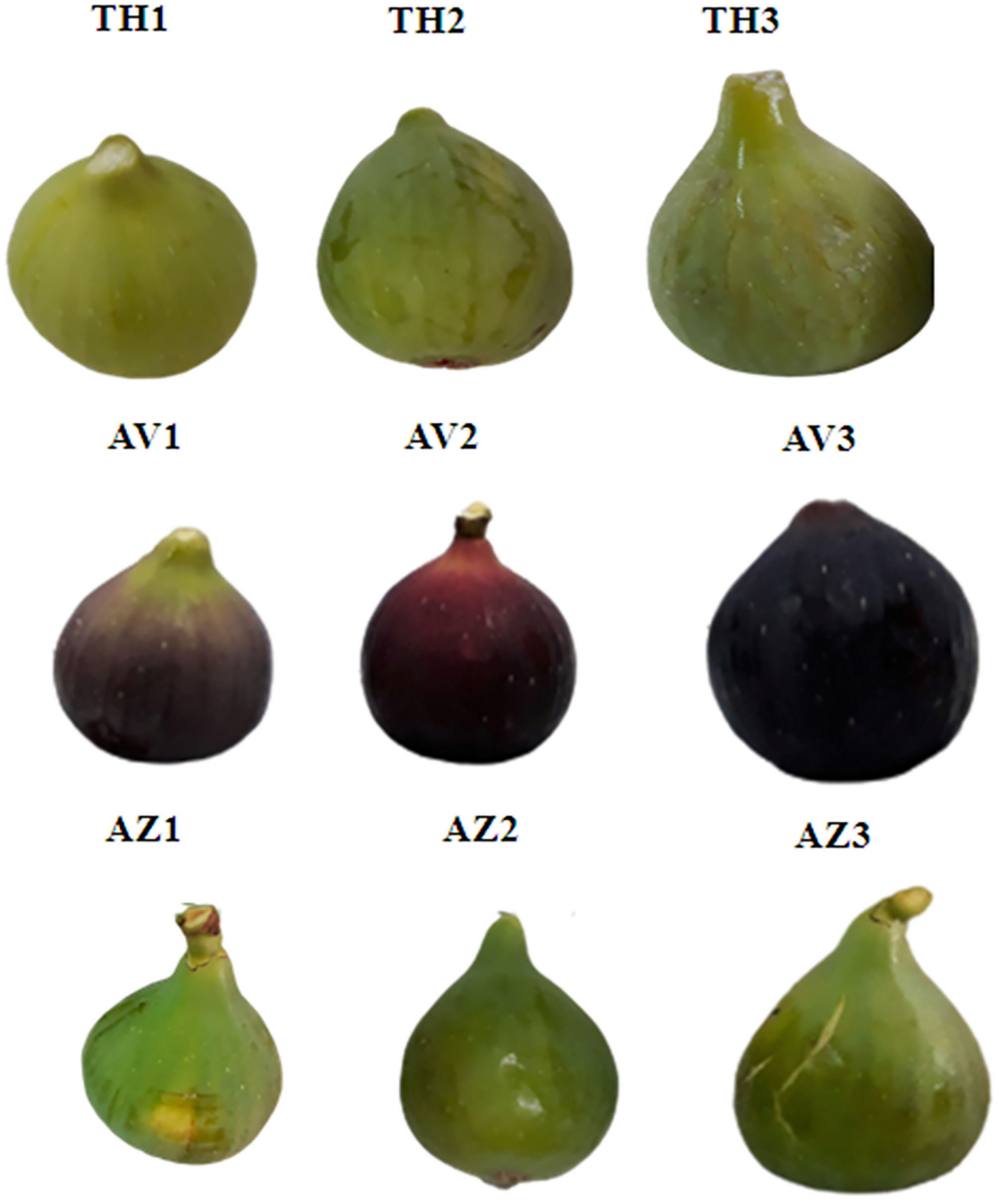
Photographs of fig cultivars at the different ripening stages. Photograph credit: Zidi Kahina, Abderrahmane Mira University. (TH) Taamriwthe, (AV) Averkane, and (AZ) Azegzaw figs; 1, unripe figs; 2, ripe figs; 3, fully ripe figs.

Fig samples were harvested in September 2018 from an orchard in Beni Maouche, Bejaia Province, in the north of Algeria. Three different fig cultivars were collected for this investigation: Taamriwthe (TH), Azegzaw (AZ), and Averkane (AV). The three ripening stages (unripe, ripe, and fully ripe) were distinguished by visual maturity characteristics based on color change and fruit firmness (Figure 1).

For TH figs, the unripe stage (TH1) was identified by its yellow color and the firmness of the fruit, whereas the ripened fruit (TH2) tended to be softer and yellow on the surface. Fully ripe (TH3) figs tended to lose their firmness and the surface cracked slightly. For AZ figs, unripe (AZ1) fruit were always firm with green-yellow skin, while ripe ones (AZ2) were soft with a dark-green color. Fully ripe figs (AZ3) became softer with cracking skin. For AV figs, unripe fruit (AV1) were identified as having purple-green skin and being hard; as they began to ripen (AV2) they slightly lost their firmness and became purple. When these figs reached the fully ripe stage (AV3), the fruit turned dark and lost its firmness.

Fig fruits were selected according to their color, size, and absence of physical damage. Then they were frozen (−20°C). Samples were homogenized with a blender (IKA A11 Basic) before analyses.

### Headspace Solid-phase Microextraction (HS-SPME)

Several methods can be used for sample comparison by SPME, including in-fiber standardization and traditional calibration methods using external and internal standards (Wang et al., 2005; Bicchi et al., 2011). In the present research, the relative contents of the identified VOCs were presented as normalized peak areas.

Optimization of the parameters affecting SPME efficiency was realized according to Pawliszyn (2000). The sample weight, salt concentration, equilibrium time, extraction time, and extraction temperature were all optimized in order to reach the linear headspace conditions.

#### SPME Fiber

A Divinylbenzene/Carboxen/Polydimethylsiloxane (DVB/ CAR/PDMS) stationary phase 50/30 μm, 1 cm length fiber was used for SPME (Supelco, Bellefonte, PA, USA). This fiber was chosen because of its efficiency in extracting a wide range of VOCs according to their molecular weight (40–275 g/mol) and polarity (Elmore et al., 2000).

The fiber was conditioned at 270°C for 30 min before first use. The equilibration step was performed in an HS-SPME vial of 20 mL containing 3 g of blended fruit samples for 25 min at 40°C. Then the fiber was exposed to the headspace for another 25 min at the same temperature for the adsorption of VOCs. In order to optimize the VOC volatility and the extraction yield, 10% of NaCl (w/w) was added with agitation (250 rpm) in the same conditions (Fiorini et al., 2015). 1,2,3-trichloropropane (3 μL) was used as an internal standard. The HS-SPME procedure was achieved using a GERSTEL MSP 2 Multipurpose Sampler (GERSTEL GmbH & Co. KG, Mülheim, Germany). The fiber was then desorbed for 1 min into the GC injector at 250°C.

#### Gas Chromatography Coupled With Mass Spectrometry

The volatile compound analyses was performed according to the method developed by Tanoh et al. (2020) with some modification, using gas chromatography coupled with mass spectrometry on an Agilent 7890A GC system (Agilent, Santa Clara, CA, USA) equipped with a 5975C Inert XL EI/CI/MSD detector at 70 eV. Volatile molecules were separated using a capillary column, VF-WAX ms 30 ×0.25 mm and 0.25 μm film thickness (Agilent Technologies, Santa Clara, CA, USA), with 1.3 mL/min at a constant flow rate of carrier gas (helium), in splitless injection mode.

**Figure 2:**
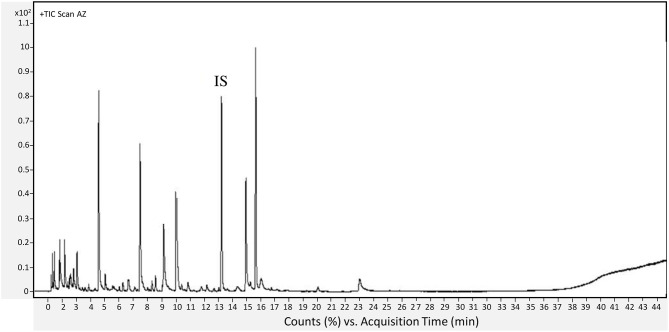
Representative chromatogram of VOCs obtained form AZ figs at unripe stage. IS, internal standard.

The oven temperature was set at 40°C for 1 min then increased by 5°C/min up to 220°C, 10°C/min to 250°C, and held at 250°C for 5 min. The temperatures of the source and the quadrupole were, respectively, 230 and 150°C, with a scanned mass between 35 and 500 amu. Each component was identified by comparing the obtained mass spectra with the Wiley (Pal 600K®) data library. Further identification was realized by calculating the retention indices (RIs) using a standard mixture of *n*-alkanes, C_7_-C_30_ 1,000 μg/mL in hexane (Supelco, Bornem, Belgium), in the same chromatographic conditions. Retention indices were determined according to the equation described by Babushok et al. (2011). The calculated RI was compared with those in the literature on the NIST, PubChem, or Pherobase databases. The data were established by Mass Hunter Workstation Software (Version B.08.00, Agilent Technologies, Inc. 2016, Santa Clara, CA, USA). Results were expressed as relative content of the identified compounds that were normalized by the internal standard. The analyses were performed on three independent biological replicates for each cultivar and each ripening stage. A representative chromatogram was presented in Figure 2.

#### Statistical Analyses

Differences in the evolution of fig volatile profiles during the three ripening stages were assessed by analyses of variance with one factor (ANOVA), performed using STATISTICA Software 7.1 (Stat Soft, Maisons-Alfort, France). Mean values were analyzed by LSD (least significant difference) at a significant level of *P* < 0.05. Principal components analysis (PCA) was analyzed using XLSTAT 5.01. A spider chart, drawn with Microsoft Office Excel 2013 (Microsoft Corporation, Redmond, WA, USA), was used to illustrate aroma profiles.

## Results

### Evolution of VOC Profile During Ripening

**Figure 3:**
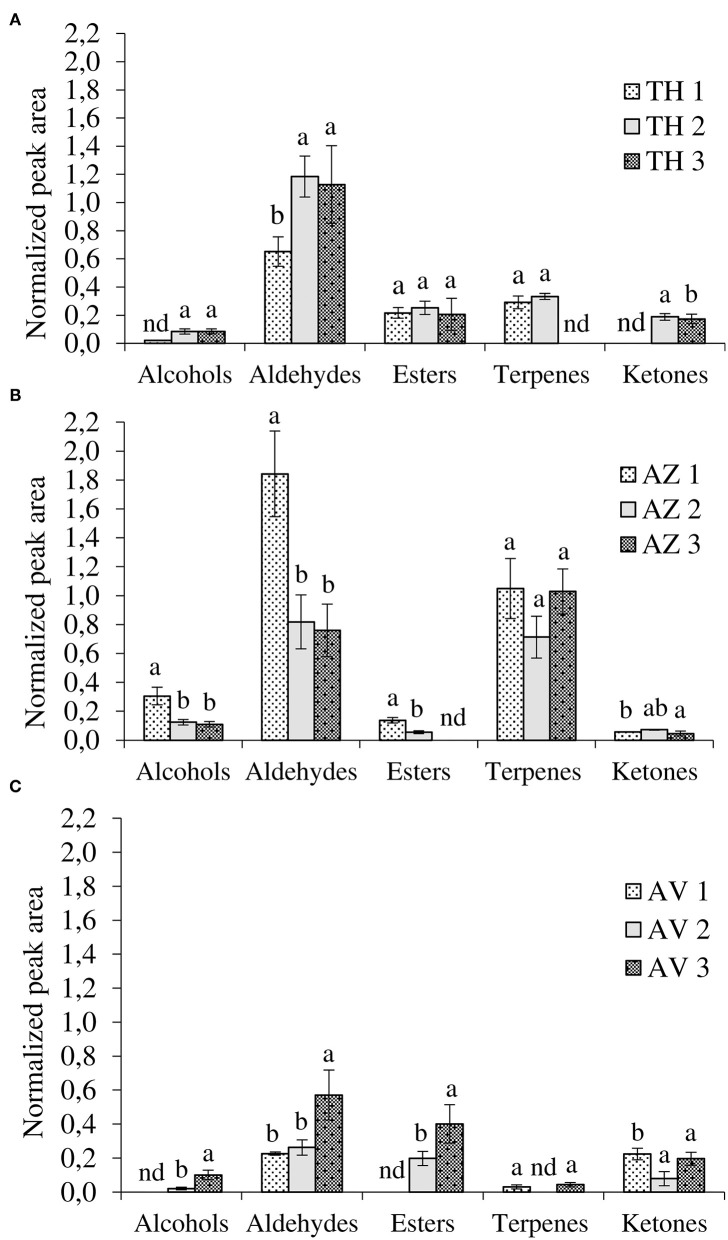
Evolution of volatile compounds during ripening according to chemical classes. **(A)** Taamriwthe, **(B)** Azegzaw, and **(C)** Averkane; 1, unripe figs; 2, ripe figs; 3, fully ripe figs; nd, not detected. Results for each VOC group from the same cultivar with different letters are statistically different (ANOVA-LSD test; *P* < 0.05; a > b).

As shown in Table 1, HS-SPME-GC/MS analyses detected 29 compounds in TH, AZ, and AV cultivars during the different ripening stages. Among these, seven alcohols, eight aldehydes, five esters, six terpenes, and three ketones were found to differ significantly, depending on cultivar and ripening stage. The evolution of VOCs during ripening for each fig cultivar is represented by chemical classes in Figure 3.

**Table 1:** Volatile organic compounds (VOCs) identified by HS/SPME coupled with GC/MS during three ripening stages of different fig cultivars.

	**Volatile compounds**	**CAS number**	**Aroma description^**A**^**	**RI^**B**^**	**RI^**C**^**	**Odor threshold in water (μg/kg)^**D**^**	**Taamriwthe**			**Azegzaw**			**Averkane**		
							**Unripe**	**Ripe**	**Fully ripe**	**Unripe**	**Ripe**	**Fully ripe**	**Unripe**	**Ripe**	**Fully ripe**
**Alcohols**															
1	Pent-1-en-3-ol	616-25-1	Pungent, like, green vegetable and tropical fruity nuances	1,155	1,155	400^1^	nd	0.034 ± 0.006	nd	0.035 ± 0.002^a^	0.026 ± 0.005^b^	0.020 ± 0.002^b^	nd	nd	0.035 ± 0.013
2	Hexan-1-ol	111-27-3	Pungent, ethereal, fruity and alcoholic, sweet with a green top note	1,348	1,347	500^2^	nd	nd	nd	0.026 ± 0.002^a^	0.024 ± 0.003^a^	0.024 ± 0.002^a^	nd	nd	nd
3	(*E*)-hex-2-en-1-ol	928-95-0	Fresh fatty green, fruity, vegetative, with leafy and herbal nuances	1,401	1,401	100^2^	nd	nd	nd	nd	0.020 ± 0.002	nd	nd	nd	nd
4	Benzyl alcohol	100-51-6	Sweet, floral, fruity with chemical nuances	1,868	1,868	10,000^3^	nd	nd	nd	0.166 ± 0.043	nd	nd	nd	nd	nd
5	Pentan-1-ol	71-41-0	Pungent, fermentedbready, yeasty, winey	1,249	1,249	nd	nd	nd	nd	nd	nd	nd	nd	0.020 ± 0.007	nd
6	3-methylbutan-1-ol	123-51-3	Fusel, alcoholic, pungent, ethereal, cognac, fruity	1,245	1,240	nd	nd	0.050 ± 0.009^b^	0.085 ± 0.017^a^	0.028 ± 0.002^a^	nd	0.037 ± 0.007^a^	nd	nd	0.032 ± 0.003
7	2-ethylhexan-1-ol	104-76-7	Citrus fresh, floral oily sweet	1,494	1,494	nd	0.020 ± 0.003	nd	nd	0.050 ± 0.011^a^	0.054 ± 0.007^a^	0.028 ± 0.005^b^	nd	nd	0.033 ± 0.011
**Aldehydes**															
8	Acetaldehyde	75-07-0	Pungent, ethereal, fresh, lifting, penetrating, fruity	nd	714	15^4^	0.124 ± 0.03^b^	0.148 ± 0.032^b^	0.273 ± 0.044^a^	0.075 ± 0.002^b^	0.064 ± 0.005^b^	0.126 ± 0.030^a^	nd	0.058 ± 0.011^b^	0.164 ± 0.043^a^
9	Heptanal	111-71-7	Fresh, green, citrus odor	nd	1,181	3^4^	nd	nd	0.032 ± 0.003	nd	nd	nd	nd	nd	nd
10	Nonanal	124-19-6	Citrus, with a fresh slightly green lemon	1,385	1,385	1–98^5^	nd	0.020 ± 0.018	nd	0.026 ± 0.007^a^	nd	0.026 ± 0.007^a^	nd	nd	nd
11	Benzaldehyde	100-52-7	Almond, fruity, powdery, nutty	1,512	1,513	350^4^	nd	0.093 ± 0.007^a^	0.075 ± 0.020^a^	0.937 ± 0.22^a^	0.424 ± 0.124^b^	0.350 ± 0.07^b^	nd	nd	0.090 ± 0.028
12	Hexanal	66-25-1	Green, fatty, leafy, vegetative, fruity and clean with a woody nuance	1,074	1,074	5^4^	0.311 ± 0.04^b^	0.553 ± 0.042^a^	0.359 ± 0.077^b^	0.788 ± 0.054^a^	0.330 ± 0.056^b^	0.258 ± 0.066^b^	0.226 ± 0.009^a^	0.152 ± 0.022^b^	0.141 ± 0.024^b^
13	(*E*)-2-methylbut-2-enal	497-03-0	Strong green fruit	1,084	1,090	nd	0.102 ± 0.031^ab^	0.157 ± 0.008^a^	0.078 ± 0.011^b^	nd	nd	nd	nd	nd	nd
14	(*E*)-hex-2-enal	6728-26-3	Green banana aldehydic fatty cheesy	1,217	1,217	17–316^5^	0.115 ± 0.001^b^	0.214 ± 0.039^ab^	0.311 ± 0.073^a^	nd	nd	nd	nd	0.050 ± 0.011^b^	0.175 ± 0.050^a^
15	(*E*)-oct-2-enal	2548-87-0	Fresh cucumber fatty green herbal banana waxy green leaf	1,421	1,421	3^6^	nd	nd	nd	0.016 ± 0.002	nd	nd	nd	nd	nd
**Esters**															
16	Ethyl acetate	141-78-6	Ethereal, fruity, sweet, grape and rum-like	nd	899	5,000^6^	0.216 ± 0.038^a^	0.159 ± 0.039^a^	0.135 ± 0.098^a^	0.137 ± 0.015	nd	nd	nd	0.198 ± 0.041^b^	0.333 ± 0.090^a^
17	Methyl hexanoate	106-70-7	Fusel, fermented, fruity, banana, ethereal	1,176	1,177	84^7^	nd	0.093 ± 0.020	nd	nd	0.035 ± 0.003	nd	nd	nd	nd
18	Methyl octanoate	111-11-5	Green, sweet, orange with vegetative nuances	1,383	1,383	nd	nd	nd	nd	nd	0.026 ± 0.002	nd	nd	nd	0.067 ± 0.022
19	Ethyl 2-methylbutanoate	7452-79-1	Fruity, and berry with fresh tropical nuances	1,041	1,041	nd	nd	nd	0.037 ± 0.008	nd	nd	nd	nd	nd	nd
20	Ethyl hexanoate	123-66-0	Sweet fruity pineapple	1,246	1,246	14^2^	nd	nd	0.034 ± 0.007	nd	nd	nd	nd	nd	nd
**Terpenes**															
21	Limonene	5989-27-5	Citrus orange fresh sweet	1,180	1,180	10^2^	nd	nd	nd	nd	0.215 ± 0.032^a^	0.109 ± 0.024^b^	nd	nd	nd
22	Linalool	78-70-6	Citrus, orange, floral	1,541	1,541	6^7^	nd	0.021 ± 0.002	nd	1.049 ± 0.18^a^	0.498 ± 0.113^b^	0.892 ± 0.120^a^	nd	nd	0.043 ± 0.013
23	Epoxylinalol	14049-11-7	Floral honey	1,728	1,729	nd	nd	0.025 ± 0.004	nd	nd	nd	0.028 ± 0.009	nd	nd	nd
24	α-Santalene	512-61-8	Woody	1,615	1,608	nd	nd	0.033 ± 0.002	nd	nd	nd	nd	nd	nd	nd
25	1,8-cineol	470-82-6	Eucalyptus herbal camphor medicinal	1,189	1,190	1,3–12^8^	nd	nd	nd	nd	nd	nd	0.030 ± 0.011	nd	nd
26	ß-caryophyllene	87-44-5	Sweet woody spice clove dry	1,579	1,579	150^1^	0.292 ± 0.045^a^	0.254 ± 0.013^a^	nd	nd	nd	nd	nd	nd	nd
**Ketones**															
27	Acetone	67-64-1	Solvent ethereal apple pear	nd	813	nd	nd	0.059 ± 0.016^b^	0.100 ± 0.017^a^	0.034 ± 0.002^a^	0.050 ± 0.004^a^	0.045 ± 0.016^a^	0.116 ± 0.016^a^	0.079 ± 0.040^ab^	0.043 ± 0.018^b^
28	Heptan-2-one	110-43-0	Cheese, fruity, ketonic, green banana, with a creamy nuance	1,173	1,173	140^4^	nd	nd	0.073 ± 0.027	nd	nd	nd	0.107 ± 0.016^b^	nd	0.152 ± 0.018^a^
29	6-methylhept-5-en-2-one	110-93-0	Fruity, apple, musty, ketonic and creamy with slight cheesy and banana nuances	1,330	1,330	160^1^	nd	nd	nd	0.022 ± 0.003^a^	0.022 ± 0.003^a^	nd	nd	nd	nd

A *Aroma description was obtained from literature data (http://www.thegoodscentscompany.com/)*;

B *RI, calculated retention indices*;

C *RI, theoretical retention indices (Pubchem, NIST, and the Pherobase); nd, not detected*;

D *All the odor thresholds were obtained from*:

1 *Tamura et al. (2001)*;

2 *Yang et al. (2019)*;

3 *Noguerol-Pato et al. (2012)*;

4 *Buttery et al. (1988)*;

5 *Buttery et al. (1969)*;

6 *Wu et al. (2016)*;

7 *Takeoka et al. (1990)*;

8 *Czerny et al. (2008)*.

*Results are expressed as normalized peak areas. Volatiles are listed in different chemical classes. Values are means ± SD of three samples analyzed in triplicate; results in the same line of each cultivar in the three ripening stages represented with different letters are significantly (P < 0.05) different (a > b > c)*.

The results showed qualitative differences between the different chemical classes depending on the fig cultivars and ripening stages. Aldehydes were the most abundant compounds in TH figs while large quantities of both aldehydes and terpenes were present in AZ figs. Aldehydes were distinctive compounds of AV figs. Moreover, significant differences in the proportion of chemical classes in the same cultivar, depending on fig ripening state, were also noted.

#### Aldehyde Profile

Aldehydes were the most abundant VOCs, demonstrated by their proportion of 50 ± 0.59%, 40.26 ± 0.27%, and 29 ± 1.20% of total VOCs for TH, AZ, and AV figs, respectively. Both TH and AV figs showed a significant increase in aldehyde content during the three ripening stages (Figures 3A,C), whereas AZ figs demonstrated a significant decline in aldehydes *(P* < 0.05). Six aldehydes were identified in cultivar TH, of which (*E*)-2-methylbut-2-enal contributes to the strong green fruit notes detected (Table 1). This aldehyde was only extracted from TH figs, and clearly decreased (*P* < 0.05) during the ripening process. During the development of TH figs the level of (*E*)-hex-2-enal increased 3-fold compared to the early ripening stage. Almost the same aldehydes were found in cultivar AZ with the exception of heptanal and (*E*)-oct-2-enal. In the case of the AV cultivar, only four aldehydes were extracted and a high content of (*E*)-hex-2-enal (31%) was detected.

Hexanal and benzaldehyde were the most abundant aldehydes in TH and AZ figs. All figs showed a significant decrease (*P* < 0.05) in hexanal during the studied stages. Acetaldehyde was identified in all fig cultivars and demonstrated a significant (*P* < 0.05) 2-fold increase from the beginning to the final stage of ripening.

#### Terpene Profile

The general trend for terpene was constant during fig ripening. The terpenes extracted from cultivar TH were present only at the ripe stage. However, ß-caryophyllene content increased significantly (*P* < 0.05) from the unripe to the ripe stage, when it was 76% higher. Limonene, epoxylinalol, and linalool were identified in cultivar AZ; it was observed that linalool was the most abundant terpene in AZ figs (87 ± 9.50%) and increased significantly (*P* < 0.05) throughout the ripening process. Only two terpenes were extracted from AV figs (linalool and 1,8-cineole). 1,8-cineole has an herbal eucalyptus odor with an odor threshold that varies from 1.3 to 12 μg/kg (Table 1).

#### Ester Profile

A significant increase (*P* < 0.05) in esters was recorded during AV fig ripening. The TH and AV cultivars had higher total esters content, with proportions of 19 ± 0.48% and 25 ± 2.34%, respectively, whereas AZ figs contained lower proportions of esters (around 3%). The esters identified differed between fig cultivars. Four esters were detected in cultivar TH; ethyl acetate was the most abundant with 76 ± 10.60% of total esters and was present at a constant proportion from the unripe to the fully ripe stage. Ethyl 2-methylbutanoate and ethyl hexanoate were only detected in TH figs and made an essential contribution to the fruity aroma (Table 1). AV figs contained a low number of esters as only two compounds were identified (methyl octanoate, and ethyl acetate). Ethyl acetate was present in large quantities and significantly increased (*P* < 0.05) during the ripening of AV figs, leading to the perception of a fruity aroma with high odor thresholds (5,000 μg/kg) (Table 1).

#### Alcohol Profile

There were significant differences (*P* < 0.05) in the alcohols produced by the three cultivars. Alcohol content significantly increased during the ripening of AV cultivars (Figures 3A,C). However, in the case of AZ figs, a significant decrease in alcohols was observed (Figure 3B).

Three different alcohols were identified in TH figs: pent-1-en-3-ol, 3-methylbutan-1-ol, and 2-ethylhexan-1-ol. The most abundant compound was 3-methylbutan-1-ol, which increased during fruit ripening. Four alcohols were extracted from the AV cultivar, among these pentan-1-ol was only identified at the ripe stage (Table 1). In the case of AZ figs, six alcohols were identified, among which were pent-1-en-3-ol and 2-ethylhexan-1-ol. Both of these compounds decreased significantly from the unripe to the fully ripe stage in AZ figs. Benzyl alcohol and (*E*)*-*hex-2-en-1-ol were only detected in cultivar AZ, and were present during the unripe and ripe stages, respectively.

#### Ketone Profile

Three ketones were identified in this study and two of them were found in all fig cultivars, with significant differences in occurrence. During the ripening period acetone significantly increased in TH figs and decreased in AV figs (*P* < 0.05). On the other hand, heptan-2-one significantly increased (*P* < 0.05) in both cultivars.

### Changes in Aroma Descriptors During Ripening

**Figure 4:**
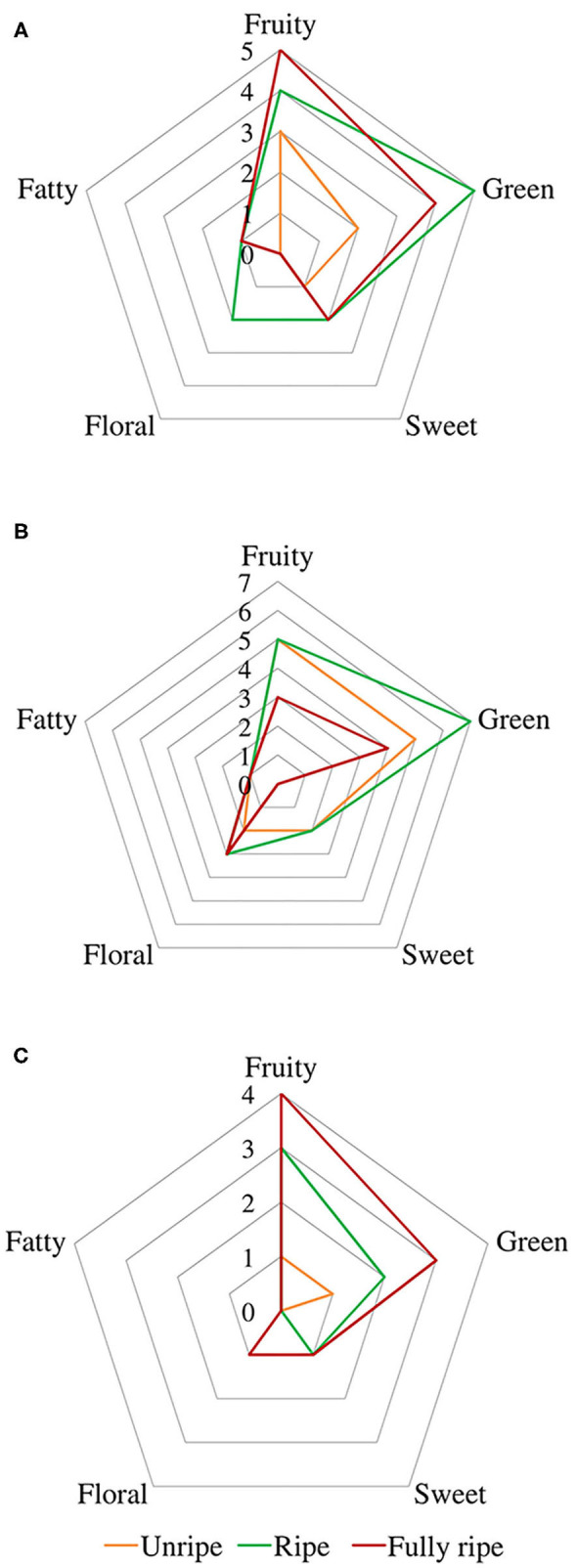
Spider chart of aromatic descriptors from fig aroma based on their odor thresholds. Cultivars **(A)** Taamriwthe, **(B)** Azegzaw, and **(C)** Averkane. The presentation consists of equiangular spokes, where each spoke representing one descriptor, and the position of the spot on the spoke was proportional to the value of the corresponding descriptor.

A spider chart representation was created using odor thresholds obtained from the literature (Buttery et al., 1969, 1988; Takeoka et al., 1990; Tamura et al., 2001; Czerny et al., 2008; Noguerol-Pato et al., 2012; Wu et al., 2016; Yang et al., 2019) and aroma descriptors (fruity, green, sweet, floral, and fatty) ^1^, represented in Figure 4, in order to better visualize changes in aroma descriptors during ripening.

**Figure 5:**
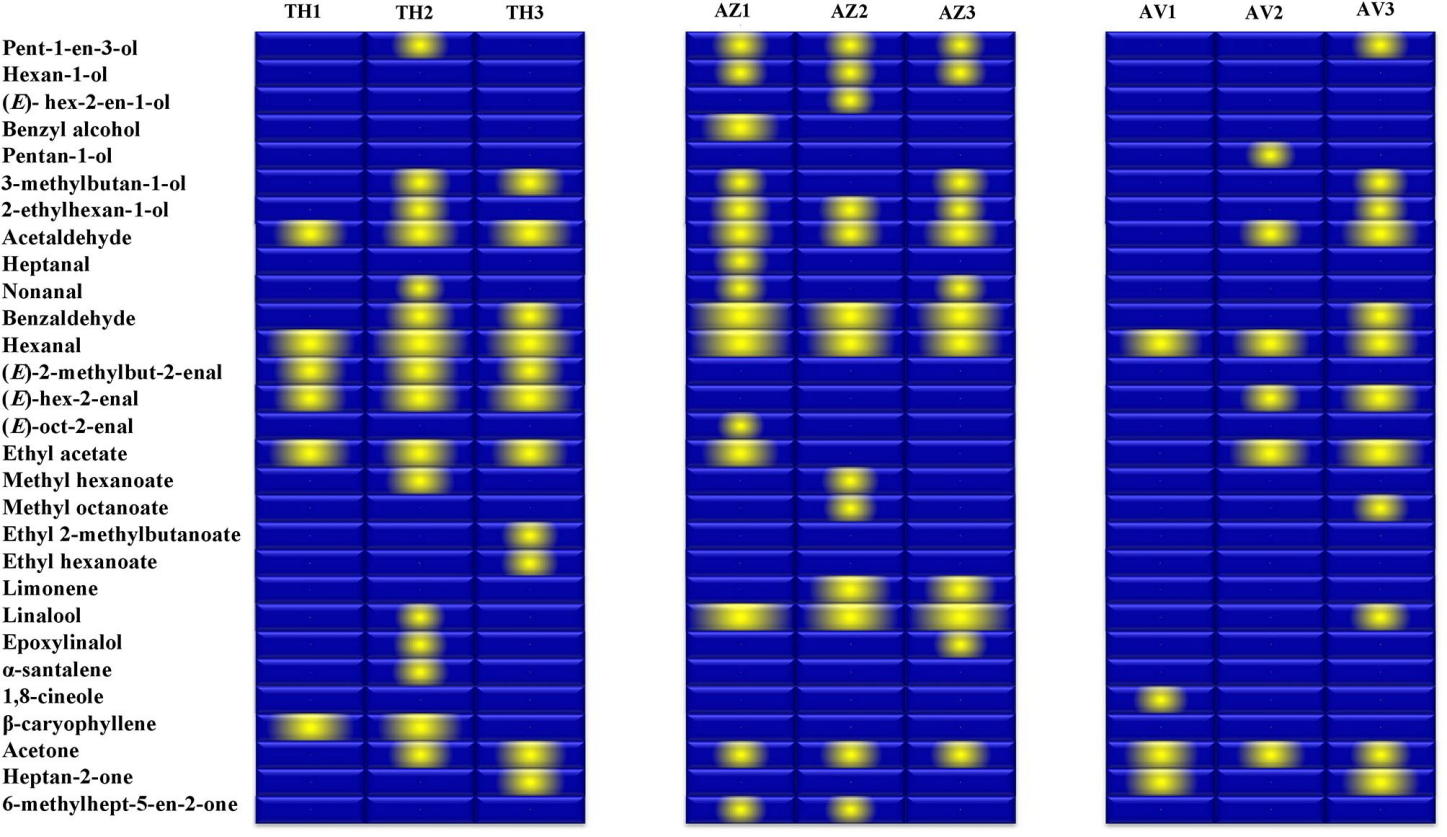
Gallery plot of VOC expression intensity of TH (Taamriwthe), AZ (Azegzaw), and AV (Averkane) figs during ripening stages. 1, unripe figs; 2, ripe figs; 3, fully ripe figs.

The gallery plot in Figure 5 presents an overview of the intensity of VOC expression for the three analyzed figs that makes it possible to differentiate between the cultivars and to produce fingerprints of the ripening stages for each fig studied. It can be observed that hexanal was the only compound identified at high relative proportion in all cultivars at all the ripening stages.

### Principal Component Analyses of Ripening Stages and Cultivar Characteristics

**Figure 6:**
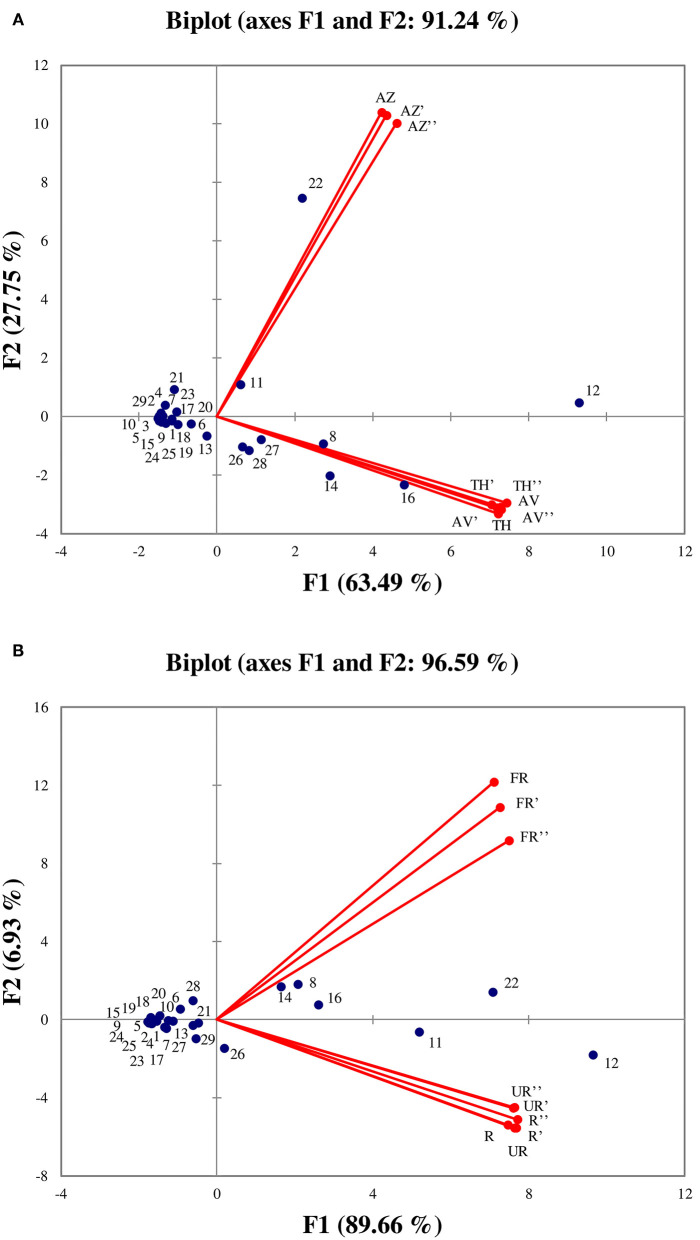
Principal components analysis performed on all identified volatile organic compounds according to the three cultivars **(A)**, and between ripening stages **(B)**. (TH) Taamriwthe, (AV) Averkane, and (AZ) Azegzaw figs. The numbers correspond to the VOCs listed in Table 1. U, unripe figs; R, ripe figs; FR, fully ripe figs.

PCA was performed in order to better visualize the VOCs that are characteristic of each cultivar as well as those determining ripening stages (unripe, ripe, and fully ripe). The PCA results showed that the two first axes explained a total of 91.24 % (F1: 63.49% and F2: 27.75%) and 96.59% (F1: 89.66% and F2: 6.93%) of the total variance (Figures 6A,B, respectively).

## Discussion

While figs are highly appreciated worldwide for their sweet taste and their high nutritional value, their smell is very weak, which makes it difficult to analyze the volatile molecules emitted by these fruits. However, as VOC analyses is a parameter of interest that is commonly used to determine the quality of fruit, it is important to develop a sensitive method to characterize fig maturation based on the most relevant volatile molecules emitted. In the present study an efficient HS-SPME-GC-MS method has been developed, which identified 29 different VOCs, belonging to five different chemical classes, in the fig samples. This result is an improvement on the two previous methods (dynamic headspace and solvent extraction), which produced only a few results when compared to the SPME technique. Twenty VOCs were identified in both TH and AZ figs, it is notable that the TH fig is a dominant cultivar in Algerian orchards because it is highly valued in both fresh and dried form. Only 14 compounds were found in AV figs, highlighting the difference in the VOC profile of the different cultivars.

Several research already focused on the study of the volatile profile of fresh figs, dried figs, fig liquors, and fig spirits (Oliveira et al., 2010; Palassarou et al., 2017; Russo et al., 2017; Rodríguez-Solana et al., 2018). Nevertheless, to the best of our knowledge, no studies involving the VOCs changes observed during the ripening process of the studied cultivars have been previously performed.

In most fig samples, aldehydes represented the most abundant VOC detected, accounting for 22 ± 1.91–38.29 ± 2.17% of the total VOCs in TH figs, from 54.37 ± 1.03 to 22.10 ± 0.43% in AZ figs, and from 21.73 ± 4.02 to 53.52 ± 5.12% in AV figs. Acetaldehyde, benzaldehyde, hexanal, and (*E*)-hex-2-enal were the most important aldehyde molecules detected; these are important aroma compounds in figs, contributing to their fresh green odor and providing a fruity flavor due to their low odor thresholds (Schwab et al., 2008; Oliveira et al., 2010; Hou et al., 2020). The high aldehyde content in all the studied cultivars could be attributed to the abundance of unsaturated fatty acid precursors (Yang et al., 2011; Garcia et al., 2012b) in figs. Indeed, C_6_ and C_9_ aldehydes can be biosynthesized from unsaturated fatty acids via the lipoxygenase pathway (Dudareva et al., 2013). Unsaturated fatty acids are oxygenated and then cleaved into C_6_ and C_9_ aldehydes by hydroperoxide lyase (Fauconnier et al., 2008; Genva et al., 2019). As an example, it is known that (*E*)-hex-2-enal is biosynthesized from linolenic acid through the lipoxygenase pathway (Garcia et al., 2012a) and that this unsaturated fatty acid represents 53% of total lipids in some fig cultivars (Jeong and Lachance, 2001). (*E*)-hex-2-enal production is augmented due to the activity of lipoxygenase on membrane lipids during fruit ripening (Karabulut et al., 2018), which explains the increase of (*E*)-hex-2-enal observed in the present study in fully ripe TH and AV figs. Similar results have been observed by Song et al. (2018) during the maturation of jujube fruit. Benzaldehyde was also highly abundant in TH and AZ figs. This molecule is one of the major aromatic aldehydes that contribute greatly to fig aroma, and it is released from the oxidation of benzyl alcohol through the shikimic acid pathway (Pereira et al., 2020). Similar abundance was reported by Palassarou et al. (2017) in the dried pulp of a Peloponnese fig cultivar. Also, a strong decrease in hexanal content was highlighted in all figs during ripening. This fall contributes to the diminution of the green aroma (Villalobos et al., 2018), and the same trend has been observed in avocado and kiwi fruit (Young and Paterson, 1995; Obenland et al., 2012). Moreover, for all studied cultivars, the acetaldehyde content increased from unripe to fully ripe figs. Pyruvate decarboxylase is an important enzyme responsible for the production of acetaldehyde, so the increase in this aldehyde can be explained by high levels of pyruvate decarboxylase enzyme activity (Echeverria et al., 2004). The increase in acetaldehyde during ripening in avocado fruits has also already been highlighted (Obenland et al., 2012) and, according to Dixon and Hewett (2000), acetaldehyde production generally occurs during the fruit ripening process. Gozlekci (2010) considers aldehydes to be among the most important chemical classes contributing to the aroma of *F. carica*. The observed decrease in total aldehydes during the later growth stage of AZ figs, compared to the other cultivars, could be due to genetic differences. Indeed, Oliveira et al. (2012) confirmed a correlation between genetic diversity and the fig metabolome. A similar decrease had already been observed by Young and Paterson (1985) and Wang et al. (2011), in kiwi fruit.

While terpenes were produced in low proportions by cultivars TH (46 ± 3.84%) and AZ (37.40 ± 1.37%), these molecules were produced in higher proportions in AV figs (58 ± 1.4%) which impacted AV figs aroma notes. Indeed, AV figs are characterized by aromatic notes provided by terpenes in comparison to the other cultivars. Terpenes are well-known for their contribution to pleasant sensory notes, as they are characterized by herbaceous, fruity, citrus, and floral scents (Arem et al., 2011; Russo et al., 2017). The molecules are synthesized by two independent pathways, the first being the mevalonate pathway, which produces volatile sesquiterpenes (C_15_). The second is the methylerythritol phosphate pathway which contributes to the production of hemiterpenes (C_5_), monoterpenes (C_10_), and diterpenes (C_20_) (Dudareva et al., 2013; El Hadi et al., 2013). During the ripening period, the terpene content of TH figs decreased markedly (46 ± 3.84–0%) while it remained constant (*P* < 0.05) in AZ (changing from 37.40 ± 1.37 to 37.06 ± 2.56%) and AV figs (changing from 41.11 ± 4.43 to 58.89 ± 10.40%) in AZ figs, which were characterized by high terpene contents. The major terpene molecule was linalool, which is recognized for its floral scents and has several biological properties (anti-inflammatory, antioxidant, and analgesic) (Aprotosoaie et al., 2014). This molecule is an important factor in the establishment of fig cultivar fingerprints, as all previous studies on fresh fig volatile molecules reported low terpene contents (Oliveira et al., 2010; Villalobos et al., 2018). The low terpene production seen in fully ripe TH figs may be due to genetic differences among the three different cultivars; for this reason, it would be interesting to perform a genomic comparison of the three cultivars studied in the present research.

Ester molecules were produced in low quantities in all the studied fig cultivars. Volatile esters are derived from the esterification of alcohols and acyl-CoA resulting from both amino acids and fatty acids catalyzed by the enzyme alcohol-*O*-acyltransferase. The low ester content of the studied cultivars could also be due to the lack of available substrate for enzymes to act on (Echeverria et al., 2004). Esters were present in low proportions in TH (29.07 ± 2.78–37 ± 0.30%) figs. However, as the total VOC content was much lower in AV figs, ester molecules accounted for a high proportion of the total VOCs in ripe (34 ± 6.18%) and fully ripe (66.5 ± 5.20%). AV figs, with quantities increasing significantly during ripening. The most abundant ester molecule, which was detected in all fig cultivars, was ethyl acetate, which is probably due to the availability of the necessary alcohol precursors (Echeverria et al., 2004). Ethyl acetate is a product of fermentation reactions and is essentially known for its pleasant fruity note at a low concentration. However, it causes an undesirable odor at higher concentrations (Rodríguez-Solana et al., 2018). The high amount of ethyl acetate (88%) in the AV cultivar could be explained by the dark-colored skin of this cultivar, which is rich in this ester and contributes to the fig aroma (Villalobos et al., 2018). Alcohol molecules were detected in most fig samples in low proportions. Alcohols (C_6_ and C_9_) are biosynthesized from unsaturated fatty acids which are oxygenated by the lipoxygenase enzyme (Deboever et al., 2020) and then cleaved into C_6_ and C_9_ aldehydes by the hydroperoxide lyase enzyme. These are then reduced into their corresponding alcohols by an alcohol dehydrogenase (Dudareva et al., 2013; El Hadi et al., 2013; Genva et al., 2019). Alcohols primarily contribute to the flowery, green, and “herby” aromas in fruits and have previously been described as pertinent flavor volatiles in fig fruits (Janzantti et al., 2012; Villalobos et al., 2018). According to our results, the alcohol content trend differed among cultivars during ripening. In this context, Wang et al. (2011) reported an increase in alcohol content during the ripening of kiwi fruit while decreasing alcohol content was observed in peaches and nectarines during ripening (Visai and Vanoli, 1997). As alcohol molecules contribute positively to the sensory appeal of fig aroma, the decrease in pent-1-en-3-ol and 2-ethyl-hexan-1-ol emission in AZ figs as they develop from unripe to fully ripe figs probably leads to a fall in green and citrus flavors during fruit ripening (Table 1).

In the present study ketones were detected at low proportions (from 21.45 ± 3.35 to 41.86 ± 3.06%) in most samples, but reached 75.93 ± 4.40% in fully ripe TH figs. The finding of small quantities of ketones in fig aroma is similar to previous studies of fresh figs by Gozlekci et al. (2011) and Oliveira et al. (2010), and with studies of both dried and fresh figs by Russo et al. (2017) and Villalobos et al. (2018). These compounds are biosynthesized from unsaturated fatty acids through the ß-oxidation pathway (Schwab et al., 2008). An increasing trend in ketone content was observed during the ripening of TH figs.

Fruity and green odors were the two dominant aroma descriptors for all fig cultivars, which showed some changes from the early to the final stage of ripening. As shown in the spider chart, there was an equivalence between fruity and green aromas in unripe AZ figs and when they reached full ripeness an equal intensity of fruity and floral notes was observed. A fatty aroma was only produced by cultivars TH and AZ, which increased with the maturity of figs (Figure 4). Sweet and floral odor characteristics were mainly present in ripe TH and AZ figs, respectively. It should be noted that certain compounds characterize the studied cultivars, such as (*E*)-2-methybut-2-enal and β-caryophyllene, which are synthesized in considerable quantities by TH figs, as well as benzaldehyde and linalool, which are released at high levels by the AZ cultivar (Figure 5).

Some volatile compounds were particularly identified at different ripening stages of each cultivar; these VOCs could be considered to represent fingerprints of the ripening stages. Among these compounds, α-santalene was exclusively identified in fully ripe TH figs, and ethyl 2-methylbutanoate and ethyl hexanoate were the two esters that characterized the fully ripe state of TH figs. In addition, the unripe AZ cultivar was distinguished by the presence of benzyl alcohol, heptanal, and (*E*)-oct-2-enal while the ripe AZ fig was distinguished by only one alcohol [(*E*)-hex-2-en-1-ol]. Moreover, the two earlier ripening stages of AV figs were characterized by 1,8 cineol (AV1) and pentan-1-ol (AV2).

As shown in Figure 6A, the AZ cultivar is mainly characterized by linalool and benzaldehyde, whereas TH and AV figs have similar VOCs, as they are characterized by acetaldehyde, (*E*)-hex-2-enal, ethyl acetate, and acetone. From examination of Figure 6A it can be concluded that the TH and AV cultivars are very similar in terms of VOC composition and differ from AZ figs. Figure 6B shows the projection of VOCs grouped by different ripening stages. The axis characterizing unripe and ripe figs have been superimposed, showing that VOC content in these two ripening stages was not significantly different. The fully ripe figs were placed the other side of the axis from the other two ripening stages, and were characterized by ethyl acetate, acetaldehyde and (*E*)-hex-2-enal.

The increasing trend seen in VOC chemical classes during ripening may be due to the climacteric nature of figs. Indeed, Lalel et al. (2003) established a relationship between the biosynthesis of VOCs and ethylene production. The difference observed between VOC class evolution during the ripening process in the different cultivars could be explained by physiological pathways specific to ripening processes in the studied cultivars.

The VOC analyses performed on the three fig cultivars at three ripening stages revealed the presence of a total of 29 compounds belonging to five chemical classes. Overall, 20 VOCs were identified in both TH and AZ figs, showing that there is no difference between the total VOC content of a dominant cultivar (TH) and one which is endangered (AZ). However, only 14 compounds were found in AV figs. Fig aromas are represented by different aromatic descriptors including high fruity, green notes and a moderate sweet and floral aroma, as well as a slight note of fatty aroma. In this investigation, we demonstrated that cultivars and/or ripening stages can be distinguished by VOCs that can be considered as specific fingerprints and are also of interest for identification in examining the agro-biodiversity of figs. Of course, further analyses is required to study the genetic and environmental factor that can affect the production of VOCs. The study of the bound aroma form of VOCs during the ripening process will be interesting of itself and help researchers to better understand the natural dynamic process of fig aroma biosynthesis.

## Data Availability Statement

The original contributions presented in the study are included in the article, further inquiries can be directed to the corresponding author.

## Author Contributions

KZ, DEK, and M-LF conceptualized the research. KZ and MB-b contributed to the data creation and performance of the software. KZ wrote the original draft and conducted the formal analyses. DEK and M-LF contributed to the methodology, supervision, and validation of work. All authors reviewed and edited the manuscript.

## Conflict of Interest

The authors declare that the research was conducted in the absence of any commercial or financial relationships that could be construed as a potential conflict of interest.

## Footnotes

^1^The Good Scents Company. Available online at: http://www.thegoodscentscompany.com (accessed Jan 30, 2020).
